# Microarray, IPA and GSEA Analysis in Mice Models

**DOI:** 10.21769/BioProtoc.2999

**Published:** 2018-09-05

**Authors:** Stephanie N. Oprescu, Katharine A. Horzmann, Feng Yue, Jennifer L. Freeman, Shihuan Kuang

**Affiliations:** 1 Department of Animal Sciences, Purdue University, West Lafayette, United States; 2 Department of Biological Sciences, Purdue University, West Lafayette, United States; 3 School of Health Sciences, Purdue University, West Lafayette, United States

**Keywords:** Microarray, Ingenuity pathway analysis, Gene set enrichment analysis, Knowledge-based software, Transcriptomic-based analysis

## Abstract

This protocol details a method to analyze two tissue samples at the transcriptomic level using microarray analysis, ingenuity pathway analysis (IPA) and gene set enrichment analysis (GSEA). Methods such as these provide insight into the mechanisms underlying biological differences across two samples and thus can be applied to interrogate a variety of processes across different tissue samples, conditions, and the like. The full method detailed below can be applied to determine the effects of muscle-specific Notch1 activation in the *mdx* mouse model and to analyze previously published microarray data of human liposarcoma cell lines.

## [Background]

Transcriptomic analysis of various cell types is crucial to elucidate the functional elements of a cell, provides insight into cell-specific characteristics and can highlight changes associated with different development or disease stages (Wang *et al*., 2009). While RNA-sequencing has become increasingly popular, the relative cost and time to analysis may be a burden. Therefore, microarray analysis is an alternative tool for comparing relative gene-expression levels between various mRNA samples (Read *et al*., 2001). Microarray is commonly used to investigate changes associated with disease states whose gene expression patterns can be inferred or have already been defined (Amaratunga *et al*., 2007). Ingenuity pathway analysis (IPA, QIAGEN) is commonly used in conjunction with large-scale omics data and provides information about pathways, genes and other signatures that may be significantly altered across different samples. Gene set enrichment analysis (GSEA) uses gene sets and characteristics that have been *a priori* associated with various diseases or pathways in order to provide biological application to the sample of interest.

The methods described below were used by Bi and colleagues to understand the effects of Notch signaling in muscle regeneration and liposarcoma, a common soft-tissue cancer type (Bi *et al*., 2016a). These methods probed the effects of myofiber-specific Notch activation in a Duchenne’s muscular dystrophy (*mdx*) mouse model and discovered that over-activation of Notch in the *mdx* mouse model displayed similar gene-expression patterns as healthy human muscle. Similarly, Bi and colleagues performed microarray analysis, IPA and GSEA to find that over-activation of Notch in mouse inguinal white adipose tissue shares signatures of human liposarcoma (Bi *et al*., 2016b). Both of these studies underscore the importance of comparative analyses when using animal models and since many microarray datasets are available online, gene set enrichment analysis (GSEA) can be used to evaluate already published datasets with respect to the investigator’s interest at relatively low cost. Discoveries such as these are imperative towards developing therapeutic targets and furthering our understanding of biological processes and how their perturbance may influence human disease.

## Materials and Reagents

- RNA isolation and cDNA synthesis
  1. 1.5 ml microcentrifuge tubes (DOT Scientific, catalog number: RN1700-GMT)
  2. Mouse (strains purchased from the Jackson lab and used in this study: *mdx* (stock# 007914) and *Adiponectin*-Cre; transgenics were in a C57BL/6J and 129S4 mixed background)
  3. Liquid nitrogen
  4. Chloroform (Sigma-Aldrich, catalog number: 288306)
  5. Isopropanol (Fisher Scientific, catalog number: S25372)
  6. TRIzol™ reagent (Thermo Fisher Scientific, catalog number: 15596026)
  7. 75% Ethanol (diluted in RNase-free water)
  8. Nuclease-free water (Thermo Fisher Scientific, catalog number: AM9916)
  9. Optional: RNase*Zap*™ RNase Decontamination Solution (Thermo Fisher Scientific, catalog number: AM9780) or other RNase decontamination solution
  10. RNaseOUT™ Recombinant Ribonuclease Inhibitor (Thermo Fisher Scientific, catalog number: 10777019)
- Real-Time Quantitative PCR
  1. LightCycler 480 96-well Multi-well Plate (Roche Diagnostics, catalog number: 04729692001)
  2. LightCycler 480 Sealing Foil (Roche Diagnostics, catalog number: 04729757001)
  3. 1.5 ml microcentrifuge tube (DOT Scientific, catalog number: RN1700-GMT)
  4. 10 mM dNTP set (Thermo Fisher Scientific, catalog number: 10297018)
  5. RNaseOUT™ Recombinant Ribonuclease Inhibitor (Thermo Fisher Scientific, catalog number: 10777019)
  6. Oligo(dT)_18_ primer (IDTDNA)
  7. M-MLV Reverse Transcriptase (Thermo Fisher Scientific, Invitrogen™, catalog number: 28025013)
    - 5x First-Strand Buffer
    - DTT
  8. SYBR Green Master Mix (Roche Diagnostics, catalog number: 4913854001)
  9. Gene-specific primers and house-keeping gene-specific primers (*i.e*., 18S ribosomal subunit) ordered from IDTDNA.
  Note: Primers used to validate microarray results and for real-time quantitative PCR in Bi et al. (2016b) are listed in Supplemental file.
- Microarray
  1. Agilent SurePrint G3 Mouse GE 8 × 60 K chip (Agilent Technologies, catalog number: G4126A; other chips can be used depending on tissue sample)
  2. Triton X-102 (Sigma-Aldrich, catalog number: X102-500mL)
  3. 100% isopropyl alcohol (Fisher Scientific, catalog number: S25372)
  4. RNeasy Mini Kit (QIAGEN, catalog number: 74104)
    - 50 RNeasy Mini Spin Columns
    - Collection tubes
    - RNase-free Buffer RLT
    - Add 10 μl β-mercaptoethanol (Sigma-Aldrich, catalog number: M6250) 1 ml of RPE buffer
    - RNase-free Buffer RPE (concentrate)
    - Add 4x volume of 100% Ethanol to buffer
    - Buffer RW1
    - RNase-free water
  5. 75% Ethanol (100% ethanol diluted in Nuclease-free water)
  6. Other microarray kit components
    - Cyanine 3-CTP and cyanine 5-CTP
    - Spike A and Spike B Mix
    - Dilution buffer
    - T7 primer
    - 5x First strand buffer
    - 0.1 M DTT
    - 10 mM dNTP mix
    - Affinity Script RNase Block Mix
    - NTP Mix
    - T7 RNA Polymerase Blend
    - Nuclease-free water
    - 5x Transcription Buffer
    - 10x blocking agent
      1,250 μl Nuclease-free water to 10x gene expression blocking agent
    - 1x HiRPM Hybridization buffer
      Equal volume 2x HiRPM Hybridization buffer to Nuclease-free water
    - Gene expression Wash Buffers 1 and 2
    - Triton X-102 (10%)
    - RNeasy Mini Kit (see components listed above)
    - Slide staining dishes
    - Slide racks

## Equipment

- RNA isolation and cDNA synthesis
  1. Balance
  2. Pipettes (1,000 μl, 200 μl)
  3. Vortex (*e.g*., Scientific Industries, model: Vortex-Genie 2, catalog number: SI0236)
  4. Centrifuge capable of reaching 16,000 *× g* and 4 °C for 1.5 ml tubes (Eppendorf, model: 5424 R)
  5. Tissue homogenizer (*e.g*., Fisher Scientific, catalog number: FB120110)
  6. Heat block capable of maintaining following temperatures for 1.7 ml micro-centrifuge tubes: 65 °C, 37 °C, 25 °C, 70 °C
  7. Water bath (*e.g*., Thermo Fisher Scientific, catalog number: TSGP02)
  8. Optional: Rotating incubator
  9. Optional: Rotator rack
- Microarray
  1. Stir bar
  2. Pipettes (1,000 μl, 200 μl, 10 μl, multi-channel pipette)
  3. Centrifuge (Eppendorf, model: 5424)
  4. Agilent Technology Surescan Microarray Scanner
  5. Hybridization oven (Agilent Technologies, catalog number: G2545A)
  6. NanoDrop™ ND-1000 UV-VIS Spectrophotometer version 3.2.1 or higher (Thermo Fisher Scientific, model: NanoDrop™ 1000, catalog number: ND-1000)
  7. Heat block capable to maintaining following temperatures for 1.7 ml micro-centrifuge tubes: 80 °C, 70 °C, 60 °C, 40 °C, 37 °C
  8. Agilent Gene Expression Two Color Microarray (Agilent Technologies, catalog number: G4140-90050)
- RNA quantification and quality control
  1. Nanodrop™ 2000c (or spectrophotometer) (Thermo Fisher Scientific, model: NanoDrop™ 2000c)
  2. Agilent Bioanalyzer 2100
- qRT-PCR
  1. Roche Light Cycler 480 PCR System for 96-well plate

## Software

1. SAS software for statistical analysis (https://www.sas.com/en_us/software/universitv-edition.html)
2. OliqoAnalvzer 3.1 (IDT) (https://www.idtdna.com/calc/analyzer)
3. GSEA (Gene set enrichment analysis) software available at: http://software.broadinstitute.org/gsea/index.jsp
4. IPA (Ingenuity pathway analysis) software available at: https://www.qiagenbioinformatics.com/products/ingenuity-pathway-analysis/
5. Agilent Microarray Scan Control (provided with current instrumentation)
6. Agilent Feature Extraction Software 12.0 (provided with current instrumentation)
7. Agilent GeneSpring GX software (provided with current instrumentation)
8. NCBI Blastn (https://blast.ncbi.nlm.nih.gov/Blast.cqi?PAGE_TYPE=BlastSearch)
9. UCSC Genome Browser, *In silico* PCR tool (https://genome.ucsc.edu/)

## Procedures

**Figure 1.**
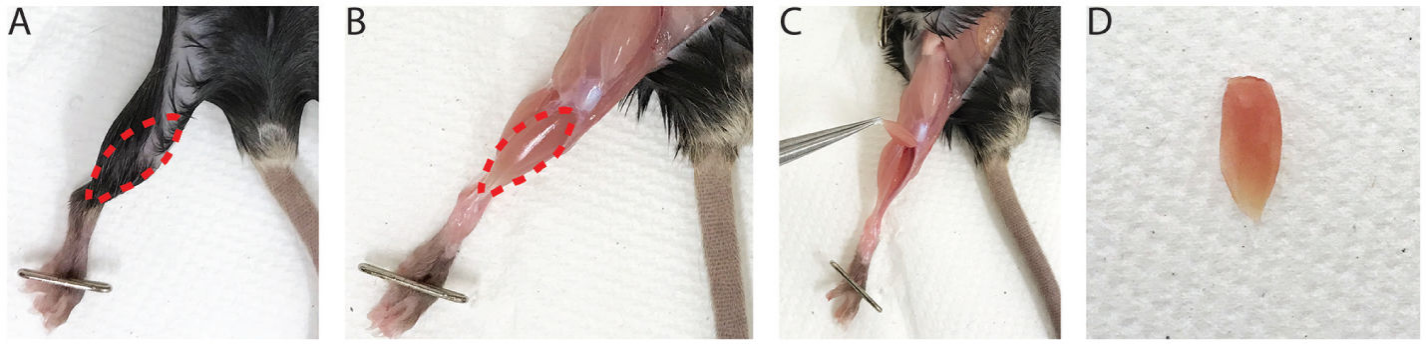
Image of tibialis anterior (TA) anatomy and dissection. Dashed red lines represent location of the TA. A. Location of TA prior to the removal of fur; B. Location of TA prior to dissection of the muscle; C. TA is cut from the tendon near the foot and pulled up towards the knee. D. Image of fully dissected TA.

**Figure 2.**
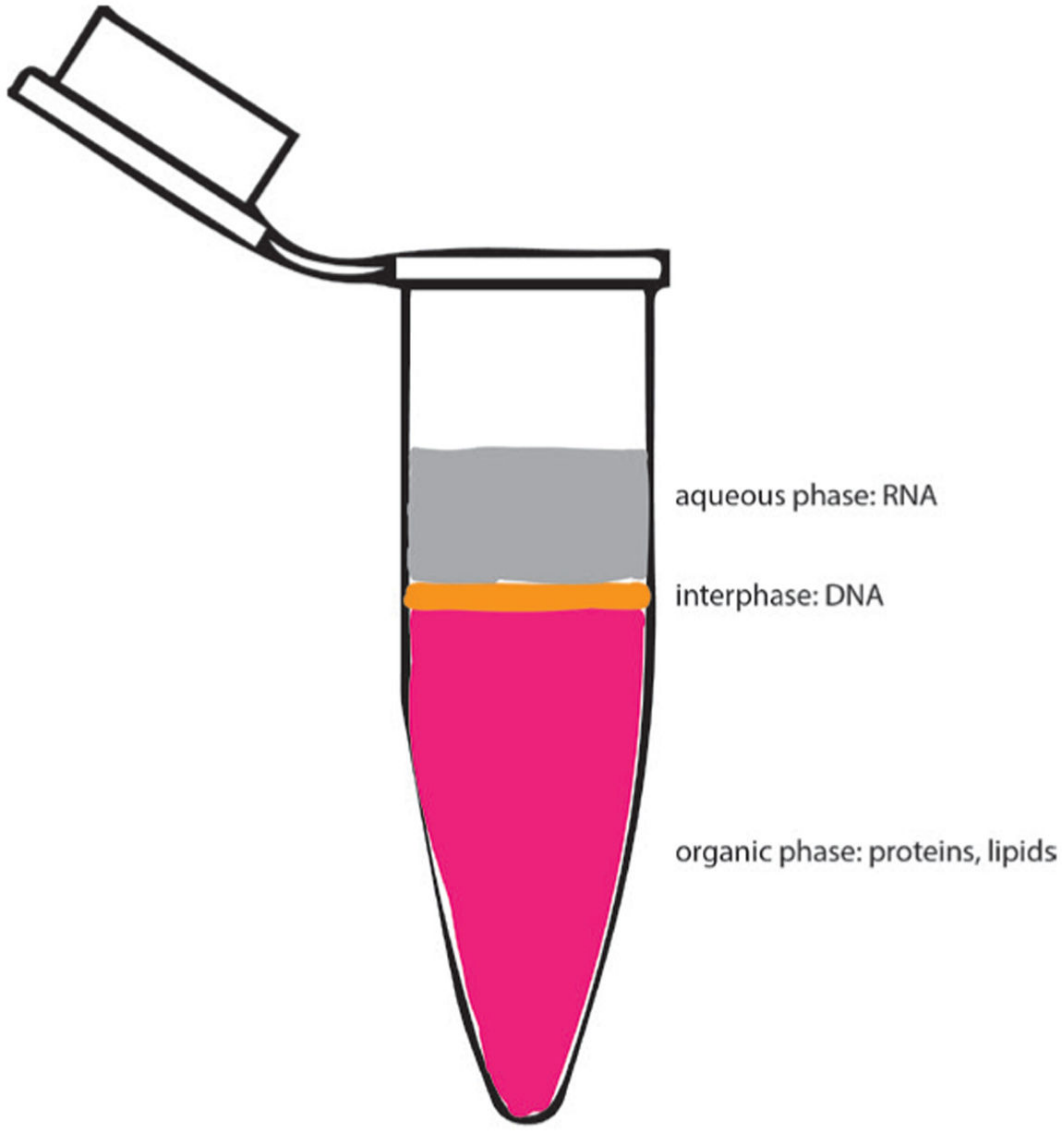
Image of phase separation following TRIzol™ reagent RNA extraction. Aqueous phase (gray) contains RNA and is to be carefully transferred into a fresh tube for downstream isolation.

**Figure 3.**
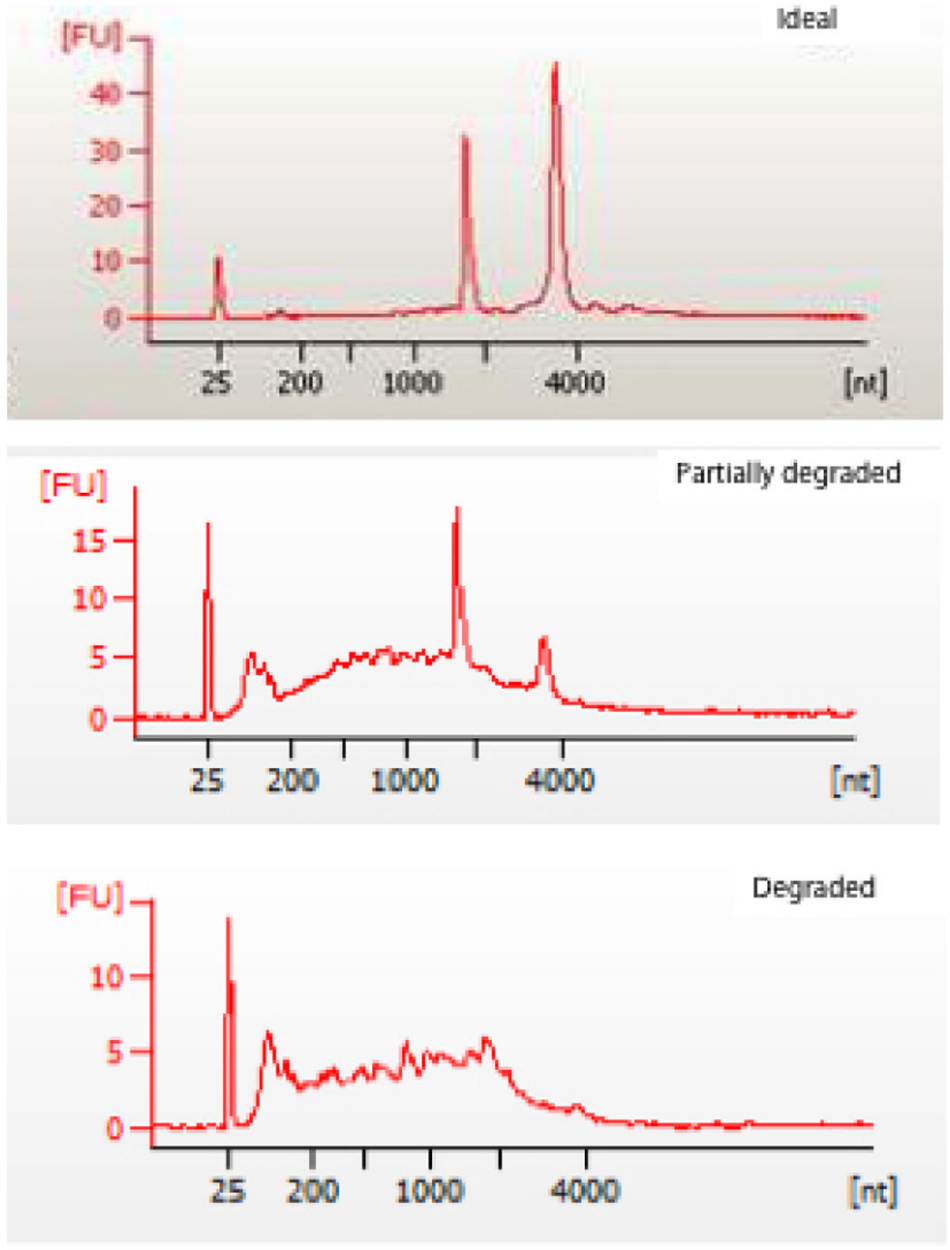
Images of intact (ideal), partially degraded and degraded RNA samples as determined by using the Agilent BioAnalyzer. The top panel clearly shows two peaks which correspond to the 18S and 28S ribosomal subunits, a small peak to a spike-in control and otherwise smooth lines. The middle and bottom panels represent degraded RNA in which the 18S and 28S peaks may or may not be clear and are usually preceded by multiple smaller peaks (indicative of degraded RNA). The x- and y-axis are nucleotides and fluorescence, respectively.

- RNA isolation
  Notes:
  - Isolate no less than 500 ng RNA from Mus musculus adult whole tibialis anterior (TA) skeletal muscle (Figure 1).
  - Maintenance of RNase-free conditions are imperative to high quantity and quality yield of RNA from tissue, and surfaces can be cleaned with RNaseZap™. RNA will be used for microarray analysis and cDNA synthesis.
  - Expect yield > 4 μg RNA/sample
  1. Dissect TA muscle or tissue of interest from respective mouse specimen (Figures 1A-1D).
  2. Weigh TA muscle, flash freeze in liquid nitrogen and add 1 ml of TRIzol™ reagent per 50-100 mg of tissue in 1.5 ml micro-centrifuge tube.
  3. Use a tissue homogenizer to homogenize the tissue for 2-3 sec at a time, placing the sample on ice to ensure it does not over-heat. The sample should be homogenized until no clear pieces of tissue are visible, approximately 2-4 times.
  4. Optional: clear lysate by centrifugation for 5 min at 4 °C and 12,000 *× g* and transfer supernatant to a new 1.5 ml microcentrifuge tube (approximate volume ~800 μl).
  5. Incubate the sample at room temperature for 5 min.
  6. Add 0.2 ml of chloroform per 1 ml of TRIzol™ reagent used, and shake or vortex vigorously for 15 sec. Then incubate the sample at room temperature for 3 min.
  7. Centrifuge for 15 min at 4 °C and 12,000 *× g* to separate phases.
  8. Transfer the aqueous phase (clear, upper phase Figure 2) containing the RNA to a new tube using a 1 ml pipette (volume ~400-600 μl), making sure not to transfer any of the interphase or phenol-chloroform phase.
  9. Add 0.5 ml of isopropanol per 1 ml of TRIzol™ reagent used to the aqueous phase containing RNA from Step A8 and incubate at room temperature for 10 min.
  10. Centrifuge sample for 10 min at 4 °C and 12,000 *× g* to precipitate RNA (RNA should form a white pellet at the bottom of the tube).
  11. Discard the supernatant being careful not to disturb the pellet.
  12. Resuspend pellet in 1 ml of 75% ethanol per 1 ml of TRIzol™ reagent used to wash the RNA pellet.
  13. Gently shake sample briefly and centrifuge for 5 min at 4 °C and 7,500 *× g*.
  14. Carefully discard supernatant, removing as much ethanol as possible without disturbing the pellet.
  15. Air-dry pellet for maximum 5-10 min at room temperature. Make sure that the ethanol has evaporated but do not let the pellet dry for too long as residual ethanol and over-drying both may affect RNA quality.
  16. Resuspend pellet in 50 μl of RNase-free water (imperative if being used for downstream microarray analysis).
  17. Measure concentration and the ratio of A_260_/A_280_ using NanoDrop Spectrophotometer.
  Notes:
  - One milligram of the sample should yield roughly 1 μg of RNA.
  - The ratio of A_260_/A_280_ in the range of 1.85-1.95 is required for downstream applications, and the RNA sample quality should be checked using the Agilent bioanalyzer.
  - Determination of the quality of RNA sample on an Agilent bioanalyzer should yield two large peaks corresponding to the 18S and 28S ribosomal subunits (Peterson et al., 2009). Examples of good and poor quality RNA are shown in Figure 3.
- Transcriptomic Microarray Analysis
  Microarray methods and analysis are adapted from the manufacturer’s manuals (Agilent, Two-Color Microarray-Based Gene Expression Analysis–Low Input Quick Amp Labeling)
  1. Preparation of Spike A Mix and Spike B Mix as positive controls, all procedures should be done in an RNase-free environment to ensure stability of RNA, following steps can be done in a 1.5 ml microcentrifuge tube.
    - Vortex and heat Spike A and B Mix at 37 °C for 5 min upon arrival and briefly centrifuge.
    - To dilute Spike A Mix (for example) prepare first dilution by adding 2 μl of Spike A Mix to 38 μl dilution buffer, mix, and briefly spin down (dilution is 1:20).
    - Dilute solution from Step B1 b by adding 2 μl of diluted solution to 78 μl dilution buffer, mix, and briefly spin down (dilution is 1:40).
    - Dilute solution from Step B1 c by adding 2 μl of diluted solution to 30 μl dilution buffer, mix, and briefly spin down (dilution is 1:16).
    - Dilute solution from Step B1 d by adding 4 μl of diluted solution to 28 μl dilution buffer, mix, and briefly spin down (dilution is 1:8).
    - Add 2 μl of final diluted solution (final dilution is 1:102,400) to 25-100 ng of sample RNA (volume should not exceed 3.5 μl).
    - Repeat for Spike B Mix for other RNA samples (*i.e*., RNA from wild-type sample vs. RNA from experimental sample) and proceed with labeling reaction.
  2. Labeling reaction, purification and quantification of fluorescently labeled complementary RNA (cRNA)
    Note: For Spike A prepare with Cyanine 3-CTP and Spike B Cyanine 5-CTP dye otherwise both samples are treated the same. Have water baths or heating blocks set to 65 °C and 80 °C prior to starting procedure for Steps B2a-B2d (below).
    - Prepare T7 primer mix by combining 1.8 μl T7 primer to 1 μl nuclease-free water per reaction.
    - Add 1.8 μl T7 primer mix to each tube and incubate at 65 °C for 10 min, gently shaking the tube every couple of minutes.
    - Remove the reactions from heat and place on ice for 5 min.
    - In the meantime, pre-warm the 5x first strand buffer at 80 °C for 3-4 min, vortex and spin down so that the buffer components are fully re-suspended.
    - Assemble the following cDNA reaction on ice in a 1.5 ml microcentrifuge tube (scaling up according to the number of reactions with one reaction in excess to correct for pipetting error):

**Table:** 

Reagent	Volume per reaction
5x First Strand Buffer	2 μl
0.1 M DTT	1 μl
10 mM dNTP Mix	0.5 μl
Affinity Script RNase Block Mix	1.2 μl
Final volume	4.7 μl
    - Add 4.7 μl cDNA reaction to each tube containing RNA + Spike Mix (final volume is 10 μl), mix by pipetting and briefly spin down.
    - Pre-heat blocks to 42 °C and 70 °C 10 min prior to use to ensure temperature reaches desired degrees (*i.e*., 42 °C and 70 °C).
    - Incubate reaction at 40 °C for 2 h then heat inactivate reaction at 70 °C for 15 min (occasionally shaking tube or using a circulating water bath).
    - Place the sample on ice for 5 min and in the meantime prepare the transcription master mix reactions (scaling up according to the number of reactions) as follows to amplify and fluorescently label the RNA:

**Table:** 

Reagent	Volume per reaction
Nuclease-free water	0.75 μl
5x Transcription buffer	3.2 μl
0.1 M DTT	0.6 μl
NTP Mix	1 μl
T7 RNA Polymerase Blend	0.21 μl
Cyanine 3-CTP (for Spike A Mix + sample) or Cyanine 5-CTP (for Spike B Mix + sample)	0.24 μl
Final volume	6 μl
    - Add 6 μl of transcription reaction to Spike A Mix or Spike B Mix (final volume per reaction now 16 μl), mix by pipetting up and down and incubate at 40 °C for 2 h.
    - Using the RNeasy Mini Kit, purify the cRNA from each reaction (protocol below as summarized per the manufacturer’s instructions)
      - Bring volume of cRNA reaction to 100 μl with nuclease-free water.
      - Add 350 μl of RLT and mix well.
      - Add 250 μl of 100% ice cold ethanol and mix by gently pipetting.
      - Transfer reaction to spin column, place in a collection tube and centrifuge at 16,000 *× g* and 4 °C for 30 sec.
      - Discard flow through and add 500 μl of RPE buffer to the column, centrifuge at 16,000 × g and 4 °C for 30 sec.
      - Repeat Steps B2j-B2k v but centrifuge at 16,000 *× g* and 4 °C for 60 sec.
      - Place column in a fresh collection tube and centrifuge at 16,000 *× g* and 4 °C for 30 sec to remove and remaining buffer.
      - Place column in a 1.5 ml microcentrifuge tube and add 30 μl of RNase-free water to the column.
      - Incubate at room temperature for 1-2 min and then centrifuge at 16,000 *× g* and 4 °C for 30 sec.
      - Optional: re-elute with eluate to increase yield.
      - Measure RNA concentration and quality with a NanoDrop™ ND-1000 UV-VIS Spectrophotometer version 3.2.1 or higher.
        1) Use ‘Microarray Measurement’ tab and select RNA-40 as sample type.
        2) Record cyanine 3 or 5 concentration, RNA absorbance ratio and cRNA concentration (RNA absorbance ratio [260/280 nm] should be 1.9 ± 0.04 and cRNA concentration should yield at minimum 1.875 μg if using 2-pack format).
  3. Hybridization of sample and probes
    - Preparation of 10x blocking: add 1,250 μl of nuclease-free water to the 10x gene expression blocking agent (supplied with the kit) and gently vortex to dissolve powder completely.
      Note: If powder does not readily dissolve, heat blocking agent at 37 °C for 4-5 min.
    - Assemble the following reaction in a 1.5 ml microcentrifuge tube.
      Note: The reaction below and subsequent volumes are for 2-pack microarray, for 1-pack, 4-pack or 8-pack refer to refer to Agilent’s Two-color Microarray-Based Gene Expression Analysis Protocol.

**Table:** 

Reagent	Volume/Mass for 2-pack microarray
Cyanine 3-labeled cRNA	1.875 μg
Cyanine 5-labeled cRNA	1.875 μg
10x gene expression blocking agent	25 μl
Nuclease-free water	Bring volume to 120 μl
25x fragmentation buffer	5 μl
Final volume	125 μl
    - Incubate at 60 °C for 30 min then place reaction on ice.
    - Add equal volume 2x Hi-RPM Hybridization Buffer to stop reaction and mix well, being careful to not introduce any bubbles.
    - Briefly spin sample down and place on ice for immediate downstream use.
    - Load gasket slide into Agilent SureHyb (Figures 4B and 4C) chamber base with label facing up.
    - Slowly add 240 μl of sample onto the gasket well from left to right, being careful not to introduce any air bubbles.
    - Make 1x solution from 2x Hi-RPM Hybridization Buffer in any wells that remain unused.
    - Place the slide with the ‘active’ side down, such that the Agilent-labeled barcode is facing down and the numeric barcode facing upwards.
    - Place the SureHyb chamber cover onto the slides, clamp both pieces and tighten.
    - Double-check that there are no stationary bubbles and, if needed, tap on surface to remove.
      Note: bubbles may be a source of artifacts as they may impact signal intensity
    - Load the chamber onto the rotator rack in the hybridization oven, set it to rotate at 10 rpm and hybridize at 65 °C for 17 h.
  4. Washing microarray slides
    - To prepare the Wash Buffers, remove outer and inner caps from container and use a pipette to add 2 ml of Triton X-102 to gene expression Wash Buffers 1 and 2.
    - Mix by inversion and replace the original outer and inner caps with the spigot provided with the kit.
    - Pre-warm gene expression Wash Buffer 2 to 37 °C before proceeding with washing the arrays.
    - Wash the staining dish prior to use as follows (repeat 2 x):
      - Add slide rack and stir bar to staining dish and fill the dish with 100% isopropyl alcohol (Figure 4A).
      - Turn on magnetic stir plate to wash for 5 min.
      - Rinse staining dish with Milli-Q water multiple times.
    - Prepare staining dishes as follows
      - Fill slide-staining dish #1 with gene expression Wash Buffer 1.
      - Place slide rack into slide-staining dish #2 and add magnetic stir bar, fill with gene expression Wash Buffer 1 and place on a magnetic plate.
      - Place dish #3 on the stir plate, add a stir bar and only fill with pre-warmed gene expression Wash Buffer 2 immediately before use.
    - Remove hybridization chamber from the rotating incubator and note any bubbles that may have formed during hybridization.
    - Disassemble the hybridization chamber by placing it on a flat surface, remove the array-gasket while maintaining the numeric barcode facing up and immediately submerge it in slide-staining dish #1.
    - Keeping the array-gasket sandwich submerged, pry open the sandwich with forceps and let the gasket slide drop to the bottom of the dish.
    - Remove the slide and place it into the slide rack in slide-staining dish #2, being careful to only touch the slide over the numeric barcode or along the thin edges.
    - Repeat these steps for the remaining slides.
    - Incubate the slide on the magnetic stir plate for 1 min.
    - Add slide rack to slide-staining dish #3 and incubate on the magnetic stir plate for 1 min.
    - Slowly and carefully remove slide rack and place slides on the slide holder.
      - Add the slide without the barcode label towards the edge.
      - Active microarray surface should be facing up towards the slide cover.
      - Close the plastic cover.
    - Proceed to scanning slides.
  5. Scanning microarray slides, feature selection and data collection
    - Place the slide holder containing slide into the scanner cassette.
    - Select the ‘AgilentG3_HiSen_GX_2color’ protocol.
    - Click’Start scan’.
    - Open Agilent Feature Extraction (FE) and add the images to be extracted to the FE project (default settings for project ok).
      Note: Manual grid mapping may be required.
    - Save the Feature Extraction project as .fep via File > Save As.
    - Select Project > Start Extracting.
- Validation of microarray results
  1. Gene-specific primer design for real-time quantitative PCR (qPCR): selection of amplicon size, primers and template are imperative to generating reproducible data that can accurately determine if the results from the microarray are validated
    Note: Pre-validated gene expression assays can be purchased from a variety of vendors and thus do not require optimization. For genes that are not available or have not been previously validated, primers efficiency should be evaluated (see below):
    - Select genes based on the microarray results. Candidate genes should be chosen based on genes that displayed a significant change across conditions and a reference gene should be chosen that did not display any change in expression across samples (for the latter examples include 18S rRNA or β-actin).
    - Amplicons targeted by primer should be approximately equal in size (not greater than 0.6 kb) and the secondary structures of the target sites can be determined using nucleic acid-folding software such as OligoAnalyzer 3.1 (IDT), as highly structured sequences can impact qPCR efficiency and results.
    - Primers target sites should be analyzed by *in silico* PCR tools such as NCBI BLAST or UCSC Genome Browser to determine specificity.
    - Each primer should have roughly the same melting temperature, however the exact ideal annealing temperature must be determined experimentally.
  2. cDNA synthesis
    - One to five microgram of template RNA from tissue samples assayed in microarray analysis required (use both biological and technical replicates here; the former being another mouse sample under the same condition and the latter the same RNA that was used for microarray analysis), and quality should be determined prior to cDNA synthesis.
    - Thaw reagents from M-MLV RT kit, vortex and centrifuge briefly.
    - Assemble the following reaction on ice in the respective order:

**Table:** 

Reagent	Volume
250 ng random primers	1 μl
10 mM dNTP mix	1 μl
Template RNA	1~5 μg
Nuclease and RNase-free water	to 12 μl
Final volume	12 μl
    - Gently flick PCR tubes to mix contents, briefly centrifuge and incubate at 65 °C for 5 min then place on ice immediately.
    - Add the following components to the reaction in the respective order:

**Table:** 

Reagent	Volume
5x First-Strand Buffer	4 μl
0.1 M DTT	2 μl
RNaseOUT (40 units/μl)	1 μl
M-MLV RT	1 μl
    - Mix reaction by pipetting up and down and incubate at 25 °C for 10 min.
    - Incubate reaction at 37 °C for 50 min.
    - Heat inactivate reaction at 70 °C for 15 min.
    - Resulting cDNA can be used immediately for real-time PCR analysis or stored at −20 °C.
  3. Real-time qPCR of candidate target genes
    Note: Important to set up biological and technical replicates as well as a negative control containing no template. 3 biological replicates (i.e., three RNA/cDNA samples from three different mice), 3 technical replicates (i.e., using the same RNA to generate cDNA) suggested per sample.
    - Primers should be re-hydrated in nuclease-free water and stored at a stock concentration of 10 pM.
    - Thaw reagents on ice and prepare the following reaction on ice (multiply the final reaction volume by the number of PCR reactions planned plus to [to account for pipetting error] create a master mix that can be aliquoted into Roche LightCycler 480 plates).

**Table:** 

Reagent	Volume
FastStart SYBR Green Master	5 μl
Forward primer (10 μM)	0.2 μl
Reverse primer (10 μM)	0.2 μl
Nuclease-free water	0.6 μl
Template cDNA (25 ng/μl)	4 μl
Final volume (per reaction)	10 μl
    - General Real-time Quantitative PCR cycler settings.

**Table:** 

Program	Temp	Time	Cycles
Initial denaturation	95 °C	10 min	1
Amplification	95 °C 60 °C 72 °C	10 sec 10 sec 15 sec	35
Melting	95 °C 65 °C Heat at 0.2 °C/sec to 97 °C	10 sec 60 sec	1
Cooling	30 °C	30 sec	1
    - C_q_ values can then be analyzed for primer efficiency and subsequent fold-change in target gene expression (C_q_ values > 35 are not recommended to use).
      Note: Samples requiring over 35 cycles may not provide reliable results and indicate that the cDNA quality or reaction efficiency is poor. While some very lowly expressed genes may yield a C_q_ value between 35-40, under those conditions it is imperative the negative control produces no signal at that cycle number

## Data analysis

**Figure 5.**
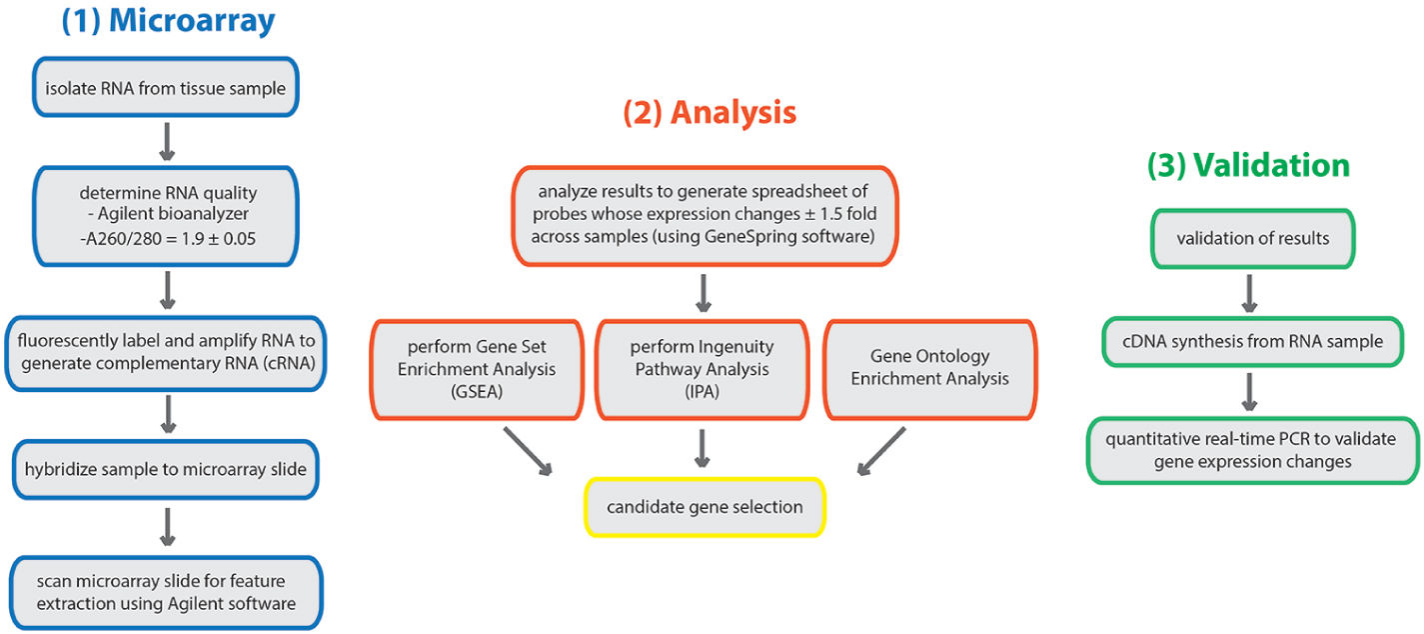
Overview of the workflow for microarray analysis. RNA is isolated from tissue samples (or cells) of interest. Quality of RNA is determined prior to proceeding with generation of cRNA, hybridization and data acquisition. Analysis of the microarray data is performed by the Agilent software. From there, the Gene Set Enrichment Analysis software is employed to yield genes that are significantly enriched in the assayed sample. Microarray data will also be used for Ingenuity Pathway Analysis (IPA) (and can be used for Gene Ontology [GO] analysis) to reveal pathways or biological processes that are enriched in the target sample.

This part of the protocol includes the analysis of the microarray results and subsequent gene-set enrichment and ingenuity pathway analyses to determine candidate genes and enriched biological pathways/processes (respectively). Following candidate gene selection, real-time PCR analysis is performed to validate candidates (for an overview of workflow see Figure 5).

- Analysis of microarray results
  Normalization, gene alignments and calls (to correlate gene expression levels) and evaluation of genes with statistically significant gene expression changes across the evaluated samples.
  1. Download GeneSpring software to perform statistical analysis and open software.
  2. Create a new project, load the text files from the feature extractor (FE) and click ‘Next’, keeping the software settings as default.
  3. The data will be uploaded onto GeneSpring upon clicking ‘Finish’.
  4. Assign ‘Experimental Grouping’ and then ‘Create an Interpretation’ with the respective experimental groupings properly selected.
  5. Statistical analysis
    - Perform an Analysis of variance (ANOVA) using the software, selecting a Tukey post-hoc test and the appropriate pairing options (depending on samples).
    - If comparing wild-type and a knock out-sample, perform a Student’s *t*-test (in general, the statistical test performed will depend on the samples).
  6. Fold-change analysis: elimination of probes that do not meet 1.5 fold-change.
    - Select ‘Fold-change’ with the same interpretation as used for the ANOVA.
    - Adjust fold-change to 1.5 and to determine how many probes meet this criterion.
    - Once ‘Finish’ has been selected, the probe lists should appear.
  7. Combine the probes whose expression increases or decreases into one excel sheet for ingenuity pathway analysis.
- Ingenuity Pathway Analysis (IPA)
  IPA is used to determine pathways that may be altered across samples based on microarray results.
  1. Login to IPA via www.ingenuity.com/products/ipa.
  2. Upload excel file that was saved from GeneSpring to software, select ‘Agilent’ and the appropriate identified type (*i.e*., appropriate species).
  3. Probe sample should be ‘ID’ and logFoldChange should be Observation 1’.
  4. Click ‘Continue’, and then use the ‘Analysis-ready’ list to select ‘Run Analysis’.
  5. Gene ontology information will then appear and can be analyzed for pathway enrichment across samples.
- Gene Set Enrichment Analysis (GSEA)
  GSEA is a computational evaluation of whether the gene expression differences across biological samples among certain gene sets reach statistical significance. This requires input of the microarray data results and selection of reference dataset for GSEA (Subramanian *et al*., 2005).
  1. Results from the microarray analysis should be filtered to yield a list of genes that display a ≥ 1.5-fold change in expression across experimental samples and reach a significance level with the corrected *P*-value of ≤ 0.05.
  2. Download GSEA software and install per the manual’s instructions (available for both R and Java).
  3. Determine the reference gene set on which the analysis should be performed: *e.g*., for Bi *et al*. (2016a) the human DMD gene expression dataset was compared to healthy human muscle which was chosen in order to understand the applicability of results to mouse models (NCBI dataset GDS3027).
  4. Microarray results containing expression data from Step C1 should be converted to the GCT file-type: Details on how to convert files to the required type for GSEA can be found on GenePattern: file format guide.
  5. A gene set database file containing a reference dataset to analyze against and a sample phenotype file must also be generated for input into GSEA (as well as an empty directory to store the output results)
    - Gene set database file (.gmt) formatting details can be found here on GenePattern: file format guide.
    - Sample phenotype file (.cls) formatting details can be found here on GenePattern: file format guide.
  6. For the analysis used in Bi *et al*. (2016a and 2016b), default settings were used when calling GSEA.
    Note: However, these can be changed as seen fit; see source code documentation for more details.
  7. GSEA results and graphical analysis
    - The output directory should contain GSEA summary results file; determine that the parameters meet the specified values prior to proceeding (ideal values can be found at Subramanian *et al*., 2005).
    - GSEA R package contains GSEA.Analyze.Sets which generates plots of the input data, refer to the source code documentation for specific parameters.
- Analysis of real-time PCR results (Bustin *et al*., 2009) (used for validation of target genes as discovered by microarray and GSEA)
  1. Primer efficiency
    - All primers used for real-time PCR should be assessed for efficiency.
    - All primers should amplify a target site with similar efficiency with respective to the reference. Efficiency of the primer amplification can be determined by generating a calibration curve.
      - Briefly, calibration curves can be determined by reverse-transcription of high-quality RNA into cDNA. cDNA is then serially diluted by a factor of 10 4-5 times (*i.e*., 1:10, 1:100, 1:1,000, 1:10,000) and the primers are assayed on each dilution.
      - Plot initial log cDNA concentration *vs*. C_q_ value
      - C_q_ = m(log cDNA concentration) + b
      - where, m = slope, b = y-intercept
      - Determine PCR efficiency
      - Efficiency is measured as 10^−1/Slope^-1
  2. Analysis of results
    - The ∆∆C_q_ is used to determine the change in expression compared to the reference gene.
      - Normalize the C_q_ value of candidate gene to reference gene (∆C_q_).
      - Transform normalized ∆C_q_ exponentially (log_2_(∆C_q_)).
      - Take the average of the technical replicates (*i.e*., three individual reactions from same biological sample) and determine standard deviation; guidelines provided in Bustin *et al*. (2009).
      - Normalize averaged results and standard deviations to reference gene.
      - Fold-change is then (1-∆∆C_q_) × 100.
    - Fold-change directionality should be consistent with results from microarray analysis.

## Notes

1. Validation of microarray results using real-time quantitative PCR
  - Validation of microarray results using real-time quantitative PCR against target genes should be done prior to GSEA since it is essential to ensure that the results obtained from the microarray can be reproduced via an alternate approach (thus substantiating their biological significance).
  - All thresholds for gene expression and real-time quantitative PCR are detailed in the above protocol. Likewise, it is imperative to have positive and negative controls, as well as biological and technical replicates in order to reach statistical significance (generally, at least three replicates/sample, however power analysis should be used to determine the sample size). For example, a negative control reaction when generating cDNA should be used for subsequent real-time quantitative PCR to confirm no contaminating materials.
  - Microarray results can be validated by real-time quantitative PCR using the same RNA used for microarray analysis. However, a power analysis should be conducted to determine the appropriate sample size for each experiment and a Student’s *t*-test with a two-tail distribution can be to analyze results unless specified otherwise.
2. Validation of microarray results using Gene-ontology (GO) term analysis
  Gene-ontology (GO) term analysis can also be performed on the list of genes generated from the microarray; however GSEA provides a rank and weight to each gene such that relative expression level in the sample is taken into consideration thus helping researchers identify candidate genes. GO term analysis does not provide gene-specific information however both GSEA and GO-term analysis will yield biological pathways that are significantly enriched in the assayed samples.

## Supplementary Material

Supplemental file

## Figures and Tables

**Figure 4.**
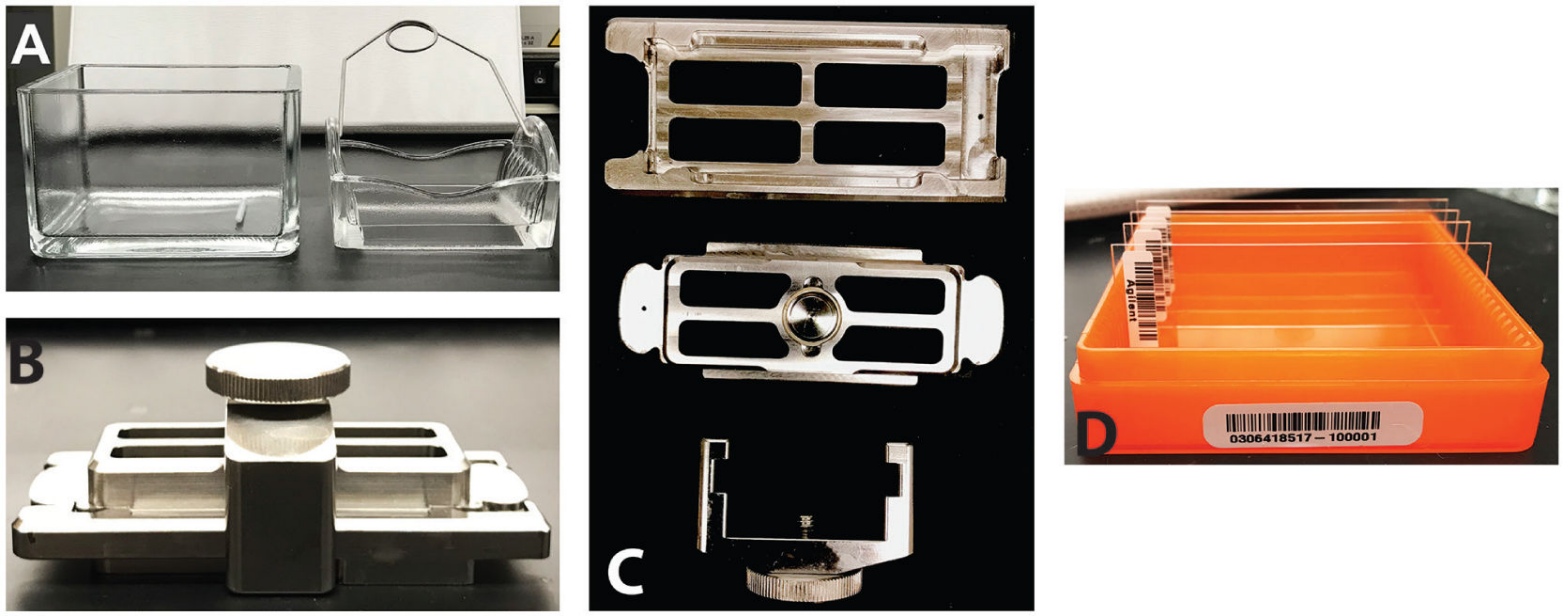
Image of microarray wash dishes, SureHyb chamber and slide. A. Image of the wash dish with metal stir bar (left) and slide holder. B. Assembled SureHyb chamber, cover and clamp. C. Individual parts of the SureHyb assembly kit. Top is the chamber, the microarray slide sits on top of it and the cover (middle) is gently placed on top. The clamp (bottom) is then used to ensure the slide and chamber stay tightly together. D. Image of microarray slides, the visible barcodes clearly state “Agilent” and are to be used for proper orientation.
