# Supplementary material for: Microarray, IPA and GSEA Analysis in Mice Models
Source: Bio Protoc. Author manuscript; Available in PMC 2018 Dec 11. (PMC6289195; doi:10.21769/BioProtoc.2999)
Supplement: Supplemental file [file NIHMS988283-supplement-Supplemental_file.docx]

**Additional Information**

| **Gene** | **Forward** | **Reverse** |
| --- | --- | --- |
| *18S rRNA* | 5′-AGTCCCTGCCCTTTGTACACA-3′ | 5′-CGATCCGAGGGCCTCACTA-3’ |
| *Notch1* | 5′-TGGCCTGCCTGTCTGGAACAACAGTTCA-3′ | 5′-ACCCTTGCCTCAGTTCAAACACAAGATAC-3′ |
| *Hes1* | 5′-CCAGCCAGTGTCAACACGA-3′ | 5′-AATGCCGGGAGCTATCTTTCT-3′ |
| *Hey1* | 5′-TGAATCCAGATGACCAGCTACTGT-3′ | 5′-TACTTTCAGACTCCGATCGCTTAC-3′ |
| *Heyl* | 5’-TCCTAGCCAGAGATTCAGTGTCAC-3′ | 5′-GTTTGTCTGCAACACCCTAGAGTG-3′ |
| *Pten* | 5′-GGAAAGGGACGGACTGGTGTAA-3′ | 5′-GCAGTGCCACGGGTCTGTAATC-3′ |
| *Lpl* | 5′-GGGAGTTTGGCTCCAGAGTTT-3′ | 5′-TGTGTCTTCAGGGGTCCTTAG-3′ |
| *Cd36* | 5′-ATGGGCTGTGATCGGAACTG-3′ | 5′-TTTGCCACGTCATCTGGGTTT-3′ |
| *Cfd* | 5′-CATGCTCGGCCCTACATGG-3′ | 5′-CACAGAGTCGTCATCCGTCAC-3′ |
| *Leptin* | 5′-GAGACCCCTGTGTCGGTTC-3′ | 5′-CTGCGTGTGTGAAATGTCATTG-3′ |
| *Cidec* | 5′-ATGGACTACGCCATGAAGTCT-3’ | 5′-CGGTGCTAACACGACAGGG-3′ |
| *Dlk1* | 5′-AGTGCGAAACCTGGGTGTC-3′ | 5′-GCCTCCTTGTTGAAAGTGGTCA-3′ |
| *Bscl2* | 5′-TGGGGCAAGAGAGACATGC-3′ | 5′-TCTTCCACAGGGACGATACCC-3′ |
| *Srebp1a* | 5′-GCGCCATGGACGAGCTG-3′ | 5′-TTGGCACCTGGGCTGCT-3′ |
| *Mdm2* | 5′-TGTCTGTGTCTACCGAGGGTG-3′ | 5′-TCCAACGGACTTTAACAACTTCA-3′ |
| *Cdk4* | 5′-AAGGTCACCCTAGTGTTTGAGC-3′ | 5′-CCGCTTAGAAACTGACGCATTAG-3’ |
| *Cdkn2a* | 5′-CGCAGGTTCTTGGTCACTGT-3′ | 5′-TGTTCACGAAAGCCAGAGCG-3′ |
| *Notch1* | 5′-GAGGCGTGGCAGACTATGC-3′ | 5′-CTTGTACTCCGTCAGCGTGA-3′ |
| *HES1* | 5′-TCAACACGACACCGGATAAAC-3′ | 5′-GCCGCGAGCTATCTTTCTTCA-3′ |
| *HEY1* | 5′-GTTCGGCTCTAGGTTCCATGT-3′ | 5′-CGTCGGCGCTTCTCAATTATTC-3′ |
| *HEYL* | 5′-GGAAGAAACGCAGAGGGATCA-3′ | 5′-CAAGCGTCGCAATTCAGAAAG-3′ |
| *Rplp38* | 5’-GAAGGAGCCAAGTCTGTCAA-3’ | 5’-GAGGGCTGGTTCATTTCAGA-3’ |
| *Pparγ* | 5’-CAAGAAACCAAAGTGCGATCAA-3’ | 5’-GAGCTGGGTCTTTTCAGAATA ATAAG-3’ |
| *UCP1* | 5’-GTGAAGGTCAGAATGGAAGC-3’ | 5’-AGGGCCCCCTTCATGAGGTC-3’ |
| *PDGFRα* | 5’-TGCTGGAACAGTGAGCCCGAG A-3’ | 5’-AGGCCACCTTCCCAGTCCTTC A-3’ |
| *AP2* | 5’-TCACCGCAGACGACAGGAAGG-3’ | 5’-CCGACTGACTATTGTAGTGTTTGA TG-3’ |
| *Pax7* | 5’-CTGCTGAAGGACGGTCACTG-3’ | 5’-GGATGCCATCGATGCTGTGT-3’ |
| *MyoD* | 5’-GGCTACGACACCGCCTACTA-3’ | 5’- CGACTCTGGTGGTGCATCTG-3 |
| *Myogenin* | 5’-TGCCCAGTGAATGCAACTCC-3’ | 5’-TTGGGCATGGTTTCGTCTGG-3’ |
